# Diagnostic performance of eNose technology in COVID-19 patients after hospitalization

**DOI:** 10.1186/s12890-023-02407-6

**Published:** 2023-04-20

**Authors:** B. F.M. van Raaij, J. D. Veltman, J. F. Hameete, J. L. Stöger, J. J.M. Geelhoed

**Affiliations:** 1 Department of Internal Medicine, Section of Geriatrics and Gerontology, Leiden University Medical Centre, Albinusdreef 2, 2333ZA, Leiden, Netherlands; 2 Department of Pulmonary Diseases, Amsterdam University Medical Centre, Amsterdam, Netherlands; 3 Department of Pulmonary Diseases, Leiden University Medical Centre, Leiden, Netherlands; 4 Department of Radiology, Leiden University Medical Centre, Leiden, Netherlands

**Keywords:** Breath analysis, COVID-19, Electronic nose technology, Follow-up

## Abstract

**Background:**

Volatile organic compounds (VOCs) produced by human cells reflect metabolic and pathophysiological processes which can be detected with the use of electronic nose (eNose) technology. Analysis of exhaled breath may potentially play an important role in diagnosing COVID-19 and stratification of patients based on pulmonary function or chest CT.

**Methods:**

Breath profiles of COVID-19 patients were collected with an eNose device (SpiroNose) 3 months after discharge from the Leiden University Medical Centre and matched with breath profiles from healthy individuals for analysis. Principal component analysis was performed with leave-one-out cross validation and visualised with receiver operating characteristics. COVID-19 patients were stratified in subgroups with a normal pulmonary diffusion capacity versus patients with an impaired pulmonary diffusion capacity (DLCOc < 80% of predicted) and in subgroups with a normal chest CT versus patients with COVID-19 related chest CT abnormalities.

**Results:**

The breath profiles of 135 COVID-19 patients were analysed and matched with 174 healthy controls. The SpiroNose differentiated between COVID-19 after hospitalization and healthy controls with an AUC of 0.893 (95-CI, 0.851–0.934). There was no difference in VOCs patterns in subgroups of COVID-19 patients based on diffusion capacity or chest CT.

**Conclusions:**

COVID-19 patients have a breath profile distinguishable from healthy individuals shortly after hospitalization which can be detected using eNose technology. This may suggest ongoing inflammation or a common repair mechanism. The eNose could not differentiate between subgroups of COVID-19 patients based on pulmonary diffusion capacity or chest CT.

## Introduction

The outbreak of the COVID-19 pandemic resulted in more than 620 million confirmed cases globally and at least 6.5 million deaths [1]. Despite improvement of treatments options, such as vaccinations, new pandemic waves caused by rapid mutations of SARS-CoV-2 still pose a great future burden on healthcare. Evidence of long-term pulmonary sequelae is emerging with persistent impaired pulmonary diffusion reported in in 23–54% of patients after 12 months depending on disease severity [2]. A systematic review of one year follow-up computed tomography (CT) in COVID-19 patients found residual abnormalities in 32.6% of patients and fibrotic-like changes in 20.6% of patients [3]. Histopathological patterns consistent with fibrosis found in multiple autopsy studies support this radiographic evidence [4]. These long-term sequelae indicate the need for a rapid and cost-effective diagnostic tool to identify COVID-19 patients with a risk of pulmonary fibrosis or persistent impaired pulmonary diffusion capacity.

Analysis of volatile organic compounds (VOCs) in exhaled breath with the use of electronic nose technology (eNose) is a relatively new and emerging diagnostic tool that already demonstrated its capability to diagnose patients with interstitial lung disease and sarcoidosis [5, 6]. VOCs produced by human cells reflect metabolic and pathophysiological processes. The VOCs found in exhaled breath after transport from blood to the lungs, thus act as biomarker of disease [7]. Furthermore, a recent study demonstrated the value of eNose technology to diagnose patients with post-COVID syndrome [8]. Analysis of exhaled breath is fast and can be easily combined with spirometry. The use of exhaled breath analysis in COVID-19 patients after hospitalization may potentially help to interpret more precisely measured serum data in combination with imaging by identifying patterns of ongoing inflammation.

We aimed to evaluate whether VOCs in exhaled breath detected with eNose technology can differentiate between previously hospitalized COVID-19 patients and healthy individuals, and discriminate subgroups with impaired lung diffusion capacity or patients with COVID-19-related CT abnormalities.

## Methods

### Study design and population

In this single-centre cross-section study, adult survivors of COVID-19 discharged from the Leiden University Medical Centre (LUMC) between March 2020 and April 2021 were invited for follow-up 3 months after discharge. Patients aged 18 years and older were eligible for inclusion after laboratory-confirmed diagnosis of SARS-CoV-2. Patients were excluded upon decline of informed consent, refusal of follow-up, a history of lung disease or inability to perform breath analysis. The study was approved by the local ethics committee for COVID-19 related research (protocolnumber 2020-059). Demographics and clinical characteristics were obtained from electronic medical records. Healthy controls were volunteers without a history of pulmonary diseases who gave informed consent for collection of their breath profile.

### Analysis of exhaled breath

Breath profiles were collected in real-time with the SpiroNose (Breathomix; Leiden, The Netherlands), a technically and clinically validated cloud-connected device that integrates spirometry and eNose technology [9]. The SpiroNose measures the complete mixture of exhaled VOCs with seven metal-oxide semiconductor sensors, each sensitive to a different group of molecules. Sensor readings are corrected for VOCs in ambient air with reading from duplicate sensors positioned on the outside of the device. Collected data is transmitted to the online and secure data platform, Breathbase, for storage, analysis and comparison with other breath profiles (ISO27001 and NEN7510 certified). Installation and usage of the SpiroNose is explained in detail elsewhere [10].

### Pulmonary evaluation

Pulmonary function tests were conducted together with the collection of breath profiles by clinical technicians at the department of pulmonary function. Forced vital capacity (FVC), forced expiratory volume in one second (FEV1) and diffusion capacity of the lungs for carbon monoxide adjusted for hemoglobin (DLCOc) were measured according local standard protocol. Impairment of lung diffusion capacity was defined as a DLCOc of < 80% of predicted. Radiological evaluation was performed with non-enhanced CT of the chest. Chest CT was defined as abnormal in patients with presence of at least one of the following COVID-19 related findings: parenchymal consolidation, ground-glass opacities (GGO), reticulation, bronchiectasis and curvilinear bands. COVID-19 patients were stratified in subgroups with a normal pulmonary diffusion capacity versus patients with an impaired pulmonary diffusion capacity and in subgroups with a normal chest CT versus patients with COVID-19 related chest CT abnormalities.

### Data analysis

Prior to analysis, sensor data was processed as described by the Vries [10]. This method creates different variables from each eNose measurement, including sensor peaks and peak/breath-hold-ratios. These variables were merged into multivariate components by principal component analysis (PCA) to avoid overfitting and decrease collinearity. Each individual eNose measurement generated 13 principal components. Retention of components was assessed visually with a screeplot and confirmed with the Kaiser criterion, which defines that a principal component with an eigenvalue of > 1 should be used in further analysis [11]. PCA plots of the first two principal components were created for visual observation of the different groups.

Breath profiles of COVID-19 patients were randomly matched with a computer algorithm for age, sex and BMI with breath profiles of healthy individuals for comparison. Between-group comparisons were analysed with Mann-Whitney U tests. Retained principal components were used as feature of linear discriminant analysis. Generated models were validated with leave-one-out cross validation and outcomes were visualized with confusion matrices and receiver operating characteristics (ROC) [12]. Area under the curve (AUC), accuracy, sensitivity, specificity, positive predictive value and negative predictive value were calculated. Statistical analysis was performed with R version 4.1.0.

## Results

In total, 167 patients with PCR-confirmed COVID-19 diagnosis were discharged from the LUMC between March 2020 and April 2021 of whom 35 (19%) patients were excluded. 22 (13%) patients had a history of lung disease and 10 (6%) patients were unable to perform breath analysis mostly due to difficulty with following PFT instructions. The remaining 135 (81%) patients were included in this analysis and their breath profiles were matched with breath profiles of 174 healthy controls.

Baseline characteristics of COVID-19 patients and healthy controls are shown in Table 1. Smoking status of healthy controls was unknown. Pulmonary function tests and chest CT are provided in Table 2.

**Table 1:** Baseline characteristics of COVID-19 patients and healthy controls

Characteristic	Patients (N = 135)	Healthy controls (N = 174)
Age, years	57 (51–67)	57 (43–65)
Sex, male	73 (54%)	93 (53%)
BMI, kg/m^2^	28.4 (25.4–32.2)	26.3 (23.9–28.4)
Hypertension	35 (26%)	6 (3%)
Diabetes mellitus	20 (15%)	6 (3%)
**Smoking status**		
Never	78 (58%)	NA
Active	2 (2%)	NA
Former	55 (41%)	NA

Data are median (IQR) or N (%). BMI = Body Mass Index. NA = not applicable.

**Table 2:** Pulmonary function and chest CT of COVID-19 patients 3 months after discharge

	3 months	N
FVC, L	3.87 ± 1.16	132
FVC, % of predicted	97 ± 19	132
FEV1, L	3.08 ± 0.87	132
FEV1, % of predicted	97 ± 18	132
Tiffeneau-index	79 ± 7	132
DLCOc, % of predicted	86 ± 18	129
DLCOc, < 80% of predicted	46 (36%)	129
Abnormal CT	96 (76%)	127

Data are mean ± SD or N (%). FVC = Forced vital capacity. FEV1 = Forced expiratory volume in one second. DLCOc = Diffusion capacity of the lungs for carbon monoxide adjusted for hemoglobin. CT = Computed Tomography. CT-SS = CT Severity Score.

### COVID-19 versus healthy individuals

**Fig. 1:**
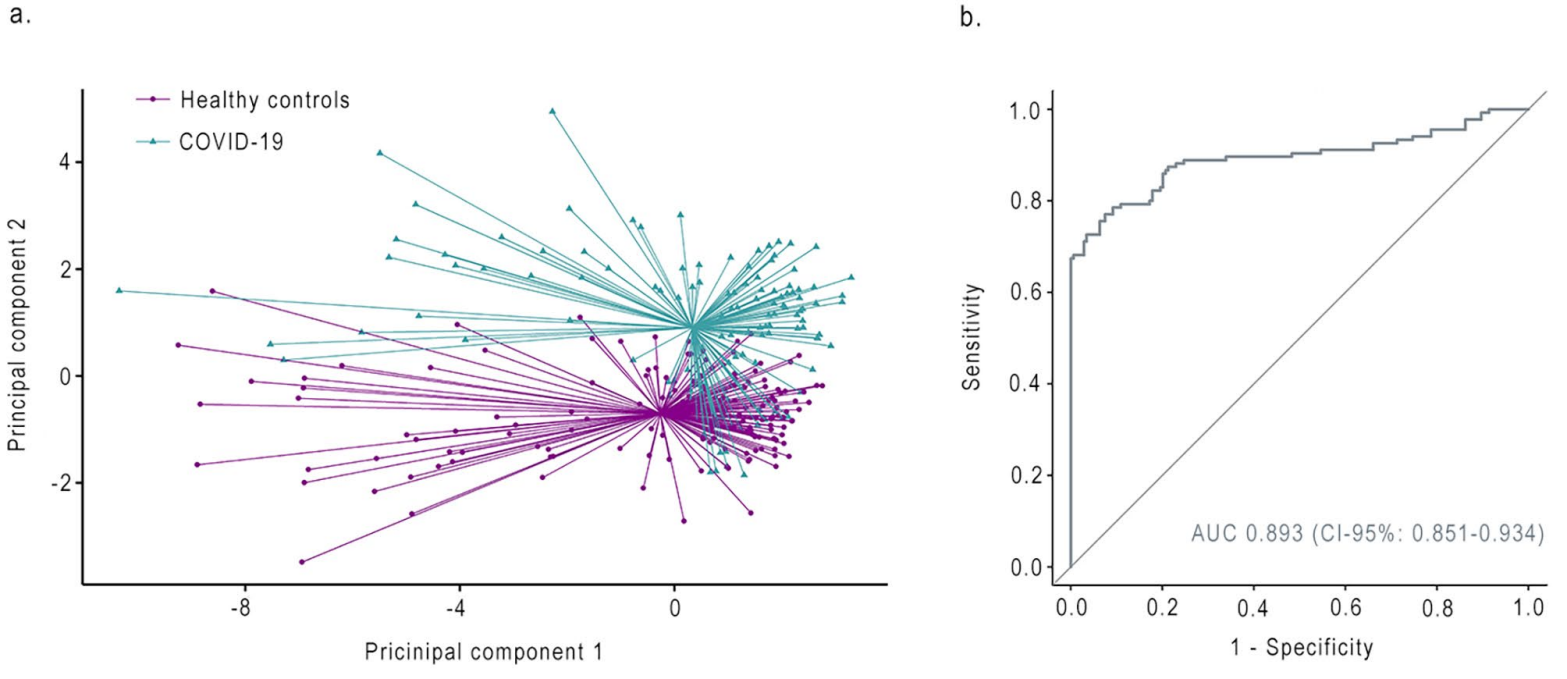
**(a)** PCA plot comparing breath profiles of COVID-19 patients after hospitalization and healthy individuals. **(b)** Receiver operating characteristic curve of principal component analysis. AUC = Area under the curve

All patients and healthy individuals were included in this analysis. The first three principle components (PCs) were retained and represented 75% of the variance. Comparison of PCs between groups resulted in p-values ranging from < 0.001–0.013 (Table 3). The eNose discriminated between breath profiles of COVID-19 patients and healthy individuals with an AUC of 0.893 (95-CI, 0.851–0.934; Fig. 1). The calculated accuracy, sensitivity, specificity, positive predictive value and negative predictive value are shown in Table 4.

**Table 3:** Results of principal component analysis

	Variance	P-value^a^
**COVID-19 versus healthy individuals**		
Principal component 1	54.4%	0.013
Principal component 2	13.0%	< 0.001
Principal component 3	7.5%	< 0.001
**COVID-19: normal lung diffusion capacity versus impaired diffusion capacity**		
Principal component 1	49.0%	0.129
Principal component 2	15.5%	0.227
Principal component 3	8.8%	0.151
**COVID-19: normal CT versus abnormal CT**		
Principal component 1	48.7%	0.796
Principal component 2	15.7%	0.880
Principal component 3	8.6%	0.818

a. Comparison of principal components between groups.

**Table 4:** Diagnostic value of eNose technology in COVID-19 patients

Group 1	Group 2	AUC (95-CI)	Accuracy	Sensitivity	Specificity	PPV	NPV
COVID-19 (N = 135)	Healthy controls (N = 174)	0.893 (0.851–0.934)	0.854	0.831	0.895	0.931	0.756
Normal lung diffusion capacity (N = 83)	Impaired lung diffusion capacity (N = 46)	0.544 (0.434–0.635)	0.678	0.778	0.672	0.152	0.976
Normal CT (N = 31)	Abnormal CT (N = 96)	0.585 (0.455–0.715)	0.740	0.000	0.752	0.000	0.979

AUC = area under the curve. 95-CI = 95% confidence interval. CT = Computed Tomography. PPV = positive predictive value. NPV = negative predictive value.

### COVID-19: normal lung diffusion capacity versus impaired diffusion capacity

This analysis included 127 patients, of whom 46 patients (36%) had impairment of lung diffusion.

**Fig. 2:**
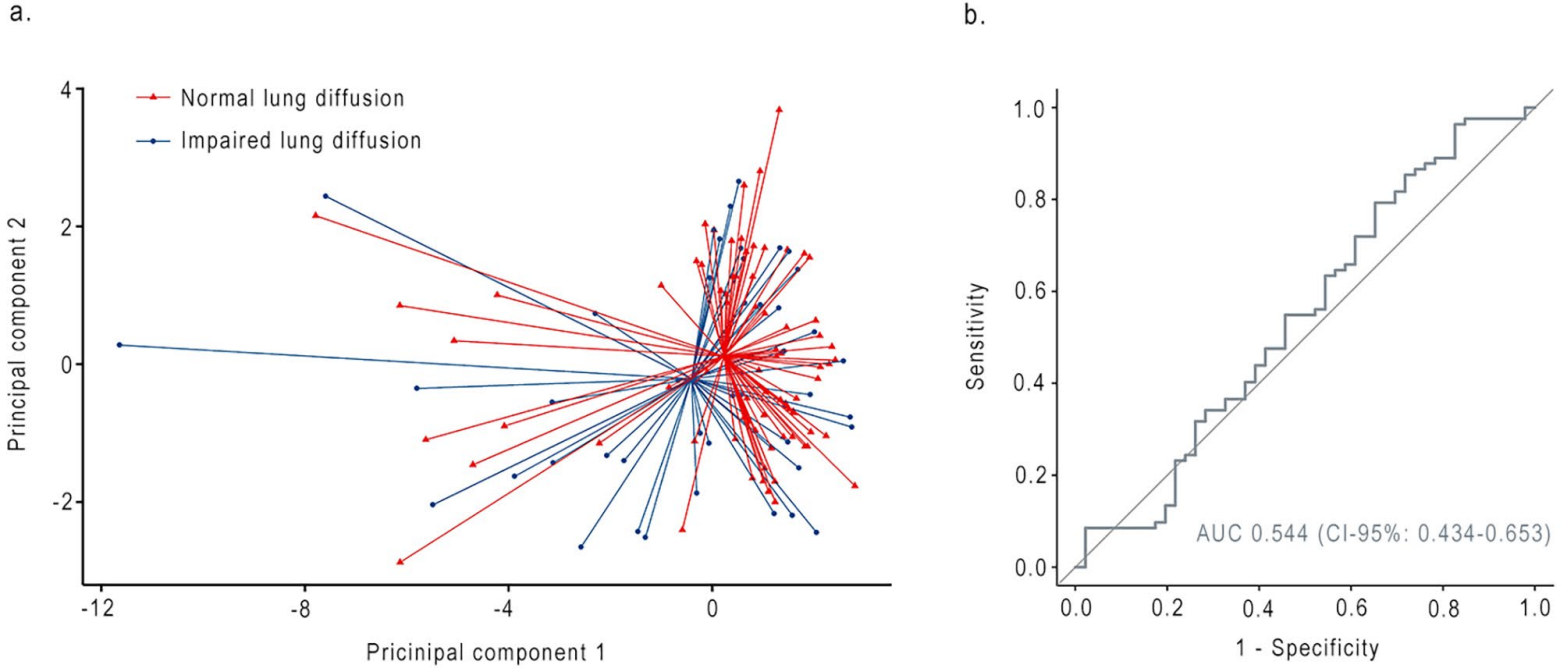
**(a)** PCA plot comparing breath profiles of COVID-19 patients with normal lung diffusion capacity and impaired diffusion capacity (defined as DLCOc < 80% of predicted). **(b)** Receiver operating characteristic curve of principal component analysis. AUC = Area under the curve

The first three PCs were retained and represented 73.3% of the variance. Comparison of PCs between groups resulted in p-values ranging from 0.129 to 0.227. Breath profiles of COVID-19 patients with impaired diffusion and patients with normal diffusion were similar with an AUC of 0.544 (CI-95, 0.434–0.653; Fig. 2).

### COVID-19: normal CT versus abnormal CT

**Fig. 3:**
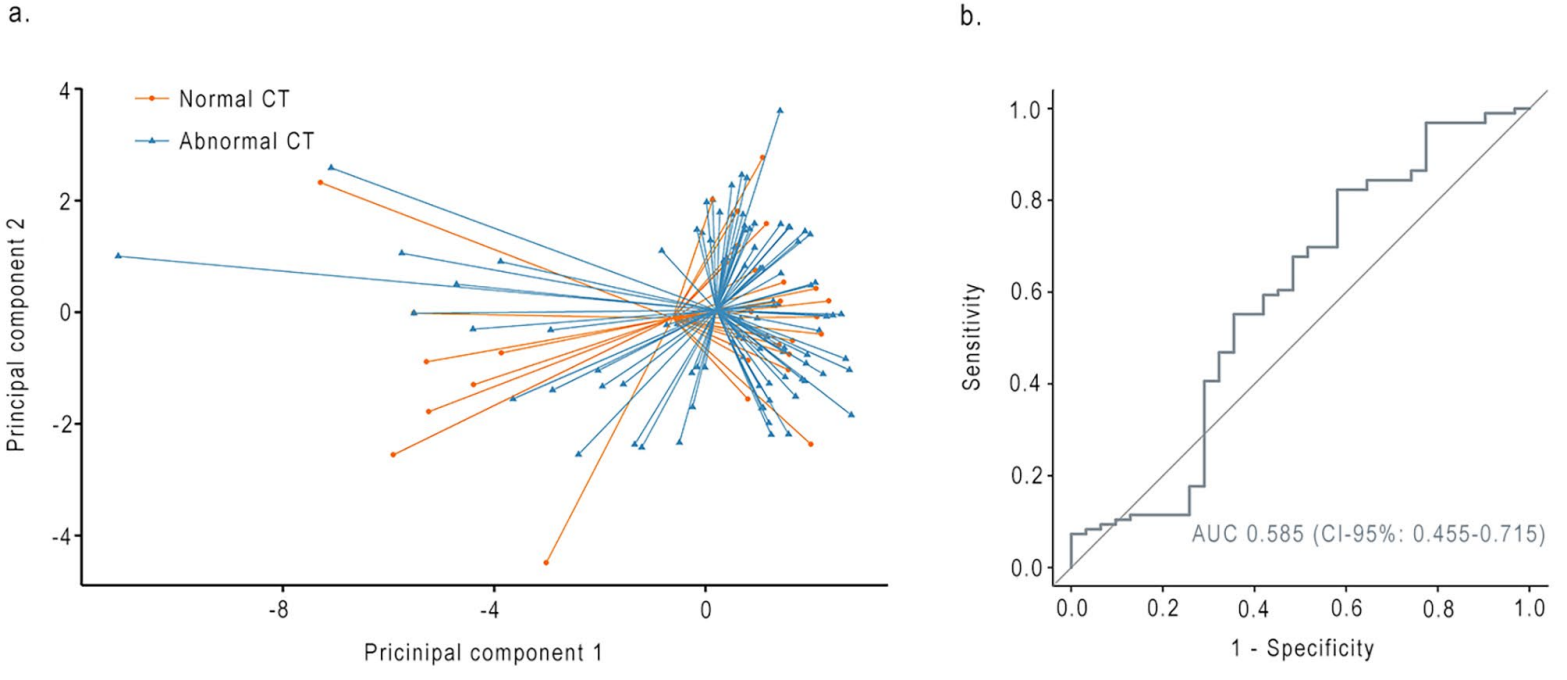
**(a)** PCA plot comparing breath profiles of COVID-19 patients with normal CT and abnormal CT (defined as presence of at least one of the following distinctive findings: parenchymal consolidation, ground-glass opacities (GGO), reticulation, bronchiectasis and curvilinear bands). **(b)** Receiver operating characteristic curve of principal component analysis. AUC = Area under the curve

This analysis included 127 patients, of whom 96 (76%) patients had an abnormal CT. The first three PCs were retained and represented 73% of the variance. Comparison of PCs between groups resulted in p-values ranging from 0.796 to 0.880. Breath profiles of COVID-19 patients with abnormal CT and patients with normal CT were similar with an AUC of 0.585 (CI-95, 0.455–0.715; Fig. 3).

## Discussion

In this study, the diagnostic performance of eNose technology was evaluated in COVID-19 patients after hospitalization with the aim to identify if VOC patterns differ from healthy controls and if breath profiles can discriminate patients with impaired lung diffusion or COVID-19 related CT abnormalities.

The eNose accurately differentiated between COVID-19 patients and healthy individuals. Within COVID-19 patients, subgroups based on lung diffusion and CT abnormalities had similar patterns of VOCs.

The diagnostic potential of breath analysis in COVID-19 patients has been investigated in different studies with a great diversity in used devices and techniques. Most of these studies compared analysis of exhaled breath with the current gold standard, detection of the SARS-CoV-2 genome from saliva samples with polymerase chain reaction (PCR) tests. Detection of SARS-CoV-2 in samples of exhaled breath is a relatively fast and cost-effective technique like eNose technology. However, the diagnostic performance of eNose devices appears to be superior with a sensitivity ranging from 81 to 100% compared to detection rates of 11–70% in studies with PCR tests of aerosolized SARS-CoV-2 [13–15]. The different types and quantities of chemo-mechanical sensors in these eNose devices appear to have comparable performances with for example a detection rate of 90% with a sensor highly selective to nitric oxide and 86% with an eNose device with carbon monoxide, nitrogen dioxide and VOC sensors [16, 17]. The use of Teraherz (THz) waves is another useful technique to detect VOCs in exhaled breath [18]. These waves are located in the electromagnetic radiation between the microwave and the infrared spectrum and the application of this technique has similar qualities as eNose technology: rapid-detection, cost-effective, easily performed and repeatable. A detailed study with THz waves demonstrated a sensitivity of 93% in differentiation of SARS-CoV-2, but more studies are needed to substantiate this result [19]. Gas chromatography-mass spectrometry (GC-MS), a technique capable of measuring VOCs and presumably also non-VOCs from exhaled breath with a maximum molecular weight of 200 Dalton, demonstrated an excellent diagnostic performance ranging from 91 to 100% accuracy [20, 21]. However, the advantage of specifying VOCs, which cannot be done with eNose technology, is outweighed by reduced cost-effectiveness, more complex operations and handling of samples and more time consuming results.

In addition to the diagnostic performance of eNose technology in the acute phase of infection, a few studies also demonstrated that COVID-19 patients with chronic complaints had a distinguishable pattern of VOCs, indicating that eNose could potentially improve follow-up assessments of COVID-19 [8]. Our study adds to this knowledge, that VOCs remain altered up to 3 months after discharge. This may suggest a mutually shared generic pathophysiological process in COVID-19 patients. The eNose could be used in addition to a COVID-19 antibody test in patients with symptoms matching post-COVID if they lack a positive PCR-test result. The difference found in VOCs between COVID-19 patients and healthy individuals could be explained by ongoing pulmonary inflammation. Normalized CRP, ESR levels and low viral load 6 weeks after hospitalization indicate recovery of systemic inflammation but do not exclude local ongoing pulmonary inflammation [22].

VOCs with the potential of being a biomarker of COVID-19 infection are yet unknown. The different cell types that play dominant roles in COVID-19 infection, like inflammatory neutrophils and monocyte-derived macrophages in the lung promote proinflammatory cytokines and chemokines with a principal role in the early cytokine storm could explain a production of different VOCs [23]. In later stages of infection, mesenchymal cells, fibroblasts and tissue macrophages are predominantly present in lung tissue and are an indication of recovery [24, 25]. This again, can lead to a production of distinguishable VOCs and the eNose could have the potential to detect changes in these inflammation-derived signals.

Although cytokines and chemokines associated with COVID-19 infection are known, it is difficult to detect them with GC-MS since their small size. Therefore, our data cannot be one-on-one correlated with prior data on cytokine and chemokine levels, but the VOCs in exhaled breath could be produced by these cells as resumed in the review article by Schulte-Schrepping J et al [24]. For example, macrophages are capable of producing all kinds of substances like nitric oxide, indoleamine-pyrrole 2,3-dioxygenase, arginase-1, reactive oxygen species and matrix metalloproteases, besides cytokines like IL-6, which can be detected in exhaled breath analysis [26]. Since eNose technology is non-invasive, it can be repeated easily as screening of actual pathophysiological processes at the site of infection. In correlation with chest CT and precisely measured cytokines this could potentially help us discriminate ongoing infection or recovery. However, in this study we did not find distinguishable VOCs in patients with or without chest CT abnormalities. This may indicate that the VOCs responsible for a change in breath profiles are common and independent of the severity of infection. Other possible explanations could be the uneven distribution of groups, the arbitrarily applied cut-off score for CT-abnormalities or a wide spectrum of different physiological processes in COVID-19 patients leading to a wide variety of VOCs which complicates differentiation of subgroups. Future studies with longer follow-up terms and preferably ILD patients as added control group are needed to investigate whether VOC signatures could serve as indication of resolution of inflammation.

A strength of this study was the selection and pulmonary evaluation of all discharged COVID-19 patients regardless of complaints or symptoms. Unfortunately, the SpiroNose is unable to present which groups of VOCs act as a specific biomarker for COVID-19. This hampers generalizability between different brands of eNose devices. This study was limited by the relatively small number of patients and uneven distribution of patients in subgroups which made it impractical to divide cohorts in a preferred training and validation set. Another limitation is the absence of patients with other lung diseases as reference group. Further studies are warranted to validate eNose technology in post-COVID-19 patients and to evaluate if these breath profiles are different compared to other lung diseases.

## Conclusions

In conclusion, eNose technology is able to distinguish between breath profiles of previously hospitalized COVID-19 patients and heathy controls. These patterns may suggest ongoing inflammation or remodelling mechanisms at play 3 months after COVID-19. Within the COVID-19 group, there were no differences in breath profiles based on lung diffusion capacity or abnormalities on chest CT.

## Data Availability

The data collected in this study is available upon reasonable request by contacting the corresponding author of this manuscript (b.f.m.van_raaij@lumc.nl). A request should include a complete analysis plan with clear hypothesis and aim of the proposed study. To gain access, data requestors will need to sign a data access agreement after approval of the request.

## Abbreviations

VOCs: volatile organic compounds
eNose: electronic nose technology
CT: computed tomography
LUMC: Leiden University Medical Centre
FVC: forced vital capacity
FEV1: forced expiratory volume in one second
DLCOc: diffusion capacity of the lungs for carbon monoxide adjusted for hemoglobin
GGO: ground-glass opacities
PCA: principal component analysis
ROC: receiver operating characteristics
AUC: area under the curve
PCR: polymerase chain reaction
THz: Teraherz
GC-MS: Gas chromatography-mass spectrometry

## References

[1] COVID-19 Dashboard. https://coronavirus.jhu.edu/map.html (accessed October 14 2022).

[2] Huang L, Yao Q, Gu X (2021). 1-year outcomes in hospital survivors with COVID-19: a longitudinal cohort study. Lancet.

[3] Watanabe A, So M, Iwagami M (2022). One-year follow-up CT findings in COVID-19 patients: a systematic review and meta-analysis. Respirology.

[4] Borczuk AC (2021). Pulmonary pathology of COVID-19: a review of autopsy studies. Curr Opin Pulm Med.

[5] Moor CC, Oppenheimer JC, Nakshbandi G et al. Exhaled breath analysis by use of eNose technology: a novel diagnostic tool for interstitial lung disease.Eur Respir J2021; 57(1). 10.1183/13993003.02042-2020 32732331

[6] van der Sar IG, Moor CC, Oppenheimer JC (2022). Diagnostic performance of Electronic Nose Technology in Sarcoidosis. Chest.

[7] Amann A, Costello Bde L, Miekisch W (2014). The human volatilome: volatile organic compounds (VOCs) in exhaled breath, skin emanations, urine, feces and saliva. J Breath Res.

[8] V RN, Mohapatra AK, Lukose VKU, Kartha J, Chidangil VB (2022). Post-COVID syndrome screening through breath analysis using electronic nose technology. Anal Bioanal Chem.

[9] de Vries R, Brinkman P, van der Schee MP (2015). Integration of electronic nose technology with spirometry: validation of a new approach for exhaled breath analysis. J Breath Res.

[10] de Vries R, Dagelet YWF, Spoor P et al. Clinical and inflammatory phenotyping by breathomics in chronic airway diseases irrespective of the diagnostic label.Eur Respir J2018; 51(1). 10.1183/13993003.01817-2017 29326334

[11] Yeomans KA, Golder PA (1982). The Guttman-Kaiser Criterion as a predictor of the number of common factors. J Royal Stat Soc Ser D (The Statistician).

[12] Smolinska A, Hauschild AC, Fijten RR, Dallinga JW, Baumbach J, van Schooten FJ (2014). Current breathomics–a review on data pre-processing techniques and machine learning in metabolomics breath analysis. J Breath Res.

[13] Subali AD, Wiyono L, Yusuf M, Zaky MFA (2022). The potential of volatile organic compounds-based breath analysis for COVID-19 screening: a systematic review & meta-analysis. Diagn Microbiol Infect Dis.

[14] Leding C, Skov J, Uhrbrand K (2022). Detection of SARS-CoV-2 in exhaled breath from non-hospitalized COVID-19-infected individuals. Sci Rep.

[15] Zamora-Mendoza BN, Diaz de Leon-Martinez L, Rodriguez-Aguilar M, Mizaikoff B, Flores-Ramirez R (2022). Chemometric analysis of the global pattern of volatile organic compounds in the exhaled breath of patients with COVID-19, post-COVID and healthy subjects. Proof of concept for post-COVID assessment. Talanta.

[16] Exline MC, Stanacevic M, Bowman AS, Gouma PI (2021). Exhaled nitric oxide detection for diagnosis of COVID-19 in critically ill patients. PLoS ONE.

[17] Wintjens A, Hintzen KFH, Engelen SME (2021). Applying the electronic nose for pre-operative SARS-CoV-2 screening. Surg Endosc.

[18] Akter N, Hasan MM, Pala N. A Review of THz Technologies for Rapid Sensing and Detection of Viruses including SARS-CoV-2.Biosensors (Basel)2021; 11(10). 10.3390/bios11100349 PMC8534088 34677305

[19] De Almeida MB, Aharonov-Nadborny R, Gabbai E (2022). Clinical trial and detection of SARS-CoV-2 by a commercial breath analysis test based on Terahertz technology. PLoS ONE.

[20] Woollam M, Angarita-Rivera P, Siegel AP, Kalra V, Kapoor R, Agarwal M. Exhaled VOCs can discriminate subjects with COVID-19 from healthy controls.J Breath Res2022; 16(3). 10.1088/1752-7163/ac696a 35453137

[21] Chen H, Qi X, Zhang L et al. COVID-19 screening using breath-borne volatile organic compounds.J Breath Res2021; 15(4). 10.1088/1752-7163/ac2e57 34624875

[22] de Graaf MA, Antoni ML, Ter Kuile MM (2021). Short-term outpatient follow-up of COVID-19 patients: a multidisciplinary approach. EClinicalMedicine.

[23] Hadjadj J, Yatim N, Barnabei L (2020). Impaired type I interferon activity and inflammatory responses in severe COVID-19 patients. Science.

[24] Schulte-Schrepping J, Reusch N, Paclik D (2020). Severe COVID-19 is marked by a dysregulated myeloid cell compartment. Cell.

[25] Liao M, Liu Y, Yuan J (2020). Single-cell landscape of bronchoalveolar immune cells in patients with COVID-19. Nat Med.

[26] Yuan ZC, Hu B (2021). Mass Spectrometry-Based human breath analysis: towards COVID-19 diagnosis and research. J Anal Test.

